# Experiences with family relationships following eating disorders: a roller coaster of emotions

**DOI:** 10.1186/s12888-025-07422-x

**Published:** 2025-10-27

**Authors:** Lisa Marie Jacobsen, Jannike Karlstad, Ragni Adelsten Stokland, Gørill Haugan, Berit Støre Brinchmann

**Affiliations:** 1 Faculty of Nursing and health Science, Nord University, Levanger, Norway; 2 Nordland Hospital of Trust, Bodø, Norway; 3 Bodø, Norway

**Keywords:** Eating disorder, Patient, Sibling, Relationship, Anorexia nervosa, Bulimia nervosa, Content analysis

## Abstract

**Background:**

Eating disorders (EDs) are life-threatening illnesses that affect both patients and their families. When a member of the family has an ED, family life is necessarily impacted, as are intrafamily relationships and communication. We aimed to shed light on patients’ and their siblings’ experiences with their family relationships during and after EDs.

**Methods:**

Our study’s sample included eight young-adult female patients previously diagnosed with anorexia nervosa (*n* = 6) or anorexia nervosa and bulimia nervosa (*n* = 2) along with two of their siblings. All patients received treatment for their EDs in 2012–2022. Data was collected in six face-to-face and four virtual individual semi-structured interviews in January–June 2024 and analyzed using qualitative content analysis.

**Results:**

Our data revealed two main themes. First, the metaphor ‘being on a roller coaster’ captured the feeling of the family living with all the highs and lows of EDs; its subthemes were strong emotions and ups-and-downs in life. Second, the metaphor ‘being in the same boat’ articulated the feeling of solidarity in the family while going through the illness period together; its subthemes were stronger attachments and open communication.

**Conclusions:**

The burden of providing care for patients with EDs can be immense and negatively impact daily life and family relationships. Family relationships affect patients, and with the intense pressure that EDs bring, it is crucial to ensure that families exert a positive influence and avoid unhelpful interactions, which may prolong the illness. Help and support with coping with EDs should be prioritized for both adult patients and their families.

**Clinical trial number:**

Not applicable.

**Supplementary Information:**

The online version contains supplementary material available at 10.1186/s12888-025-07422-x.

## Background

Eating disorders (EDs) are life-threatening illnesses that are characterized by a preoccupation with body weight and shape and involve dysfunctional eating patterns [1, 2]. Anorexia nervosa (AN) is the ED with the highest mortality rate [3]. Research has shown that EDs and psychopathological symptoms among female adolescents can be associated with problematic family functioning [4–6]. While sociocultural, psychological, genetic, and developmental factors often play a role in the development of EDs [7, 8], no causal relationship between family characteristics and EDs has been found. Moreover, although patients with EDs have reported having worse family functioning than controls, evidence indicating typical patterns of dysfunction due to EDs remains slim [36]. Even so, family interactions and relationships may be sources of additional vulnerability in the development of EDs [9], and continued engagement in unhelpful interactions can contribute to perpetuating EDs [10–12]. Poor family functioning may also exacerbate symptoms of EDs by creating a stressful environment for patients [13], and patients with EDs often end up living with their primary families longer than their peers [14–16].

EDs may lead to lower quality of life not only for patients but also for their families [17–20]. The caregiving burden can be immense, especially when combined with the negative impact of EDs on daily life and intrafamily relationships [21–24]. In the balance of receiving support and practicing self-care, parents’ aspirations can shift from fixing the ED to prioritizing a relational goal of unrelenting connection with their child [25]. A previous study underscores the importance of supporting caregivers throughout the treatment process [26]. Siblings in families with EDs have reported negative changes in family life and described how the illness takes up considerable time and space [27–29]. Siblings often do not want to burden their parents [30] and thus assume the role of caregiver, which gradually leads to exhaustion and a need for distance from the sibling with an ED in order to be able to care for themselves [31].

When a member of the family has an ED, intrafamily relationships and communication may become characterized by fear and hopelessness [32]. Including family in therapy can help participants overcome the isolation and stigma often associated with EDs [33, 34], which helps families become better at talking to each other [20]. For those reasons, help and support in coping with an ED should prioritize helping patients as well as their families [35].

The base of evidence on adolescents with EDs and families is increasing [37]; however, information regarding adult patients/former patients and their siblings reflecting back on their experiences as emerging adults on how the ED affected the intrafamily relationships is limited. This knowledge is important to better understanding how to assess future adult ED patients and their families. Our study contributes by shedding light on patients’ and siblings’ experiences with their family relationships during and after EDs. To that end, we posed the following research question for our study: *What are the experiences of patients with EDs and their siblings when it comes to intrafamily relationships?*

## Methods

### Study design

Utilizing a qualitative design, we collected data via semi-structured interviews and analyzed the data using qualitative content analysis [38].

### Participants and setting

All patients who participated in our study had been treated for EDs at a hospital in Norway between 2012 and 2022 and have, together with their families, participated in multi-family therapy (MFT) for adults offered at the same hospital [17, 20]. MFT is a comprehensive term encompassing various approaches that unite multiple families facing the same concern; it was developed for individuals with EDs, and it brings together several families with a family member with an ED. It aims to expand communication and interaction within families, as well as to empower patients to take responsibility for their own lives and health, toward independence, and reduce ED symptoms.

All patients were young women who were 18–30 years old when they were treated for a *DSM-IV*-based diagnosis of AN or bulimia nervosa (BN). Inclusion criteria for patients were: [1] not having established their own family [2], having an ongoing relationship with their family of origin [3], residing within the health region catchment area [4], having ongoing contact with a therapist in specialist health services who could provide follow-up when necessary, and [5] having participated in MFT [17]. In total, 47 patients were asked to participate, of whom eight consented. Patients also asked their siblings whether they wanted to participate, and two siblings consented. The siblings included were related to patients included in the study.

### Data collection

In January 2024, each prospective patient received a digital letter from the hospital detailing the study’s aim and asking their informed consent to participate in the study. Interested patients signed the informed consent form before the interview, and the form was subsequently stored in a locked office. The interviews were developed together with and pilot-tested by the co-researcher (RAS) in advance. Findings in previous research were used to develop the interview guide [20]. As detailed in Table 1, data were collected through individual semi-structured interviews, conducted either face-to-face (in the participants’ hometowns) or virtually (on Microsoft Teams) by the first author (LMJ) in the first half of 2024 at a time and place convenient to the participants [39]. Participants were informed that the interview would last approximately 1 h. If the participants needed someone to talk to after the interview, they could contact the project leader and get a chance to talk with a health care worker at the hospital; to the best of our knowledge, none of the participants needed this. The overall focus in interviews was: *What are the experiences of patients with EDs and their siblings when it comes to intrafamily relationships?*

**Table 1:** Overview of interviews and participants

No.	Patient or sibling (sex)	Age in years	Diagnosis	Recovery status at interview time	Accommodation status (during illness)	Interview mode	Length of interview in minutes
1	Patient	38	AN and BN		Living alone	Face to face	44
2	Patient	29	AN		Living with family of origin	Face to face	47
3	Sibling (female)	36			Living with own family	Face to face	25
4	Sibling (male)	33			Living with family of origin	Face to face	46
5	Patient	28	AN	Still in treatment	Living with family of origin	Virtual	40
6	Patient	26	AN		Living with family of origin	Face to face	49
7	Patient	23	AN and BN		Living with family of origin	Face to face	60
8	Patient	21	AN		Living with family of origin	Virtual	55
9	Patient	20	AN	Still in treatment	Living with family of origin	Virtual	46
10	Patient	21	AN	Still in treatment	Living with family of origin	Virtual	58

The total length of interviews was 470 min, and the total length of transcribed material from the interviews was 210 pages

*AN* Anorexia nervosa, *BN* Bulimia nervosa

## Analysis

Prior to describing the process for analysis, it is important to contextualize the authors. We are all women of different ages; we are two nurses, one physiotherapist, and one social worker with clinical experiences in this field. Some are mothers, and some have experience with being a family member of someone with an ED. Authors 2, 3, and 5 are professors who are well-experienced with teaching and research. RAS has experienced having an ED, and her ability to view the results from a different angle based on her experience was viewed as a valuable contribution to our study and a benefit for its internal validity (i.e., rigor); furthermore, involving patients can encourage democratic rights and improve medical knowledge [40].

All interviews were recorded, transcribed, and then analyzed by LMJ using qualitative content analysis [38], to get an overview of the field and to structure and identify patterns across the data. The interviews were recorded with a digital recorder, then transcribed verbatim by the first author as soon as possible after the interview. Memos during the interview were used to capture immediate reflections, and a summary note was written after the interview to ensure that any specific atmosphere or body language was documented.

In searching for content in the transcripts that would address our study’s aim, we followed an inductive, text-driven approach. We identified patterns by sorting content according to differences and similarities [41]. We also looked for latent content and the meaning behind the participants’ words, not only what they literally stated [41]. The processes of coding, condensation, and abstraction were performed by all authors in collaboration, to develop categories and themes leading to the generation of knowledge and patterns from the data [38]. Specifically, each interview transcript was first read through by LMJ to gain an overall impression. Meaningful units were identified inductively in Nvivo [42]. The identified units of meaning or segments of text were assigned preliminary codes (Table 2); these codes were short phrases or words that described the essence of the data in a simple form. This step was followed by categorization, where related codes were grouped into themes and subsequently condensed into shorter descriptions; all of these were reflected on, interpreted, and abstracted into subthemes and later into themes describing their underlying meaning [41]. The analysis was a nonlinear back-and-forth process, moving between the whole text and the condensed descriptions, and saturation was reached when no new insights were found.

**Table 2:** Example of qualitative content analysis by Granheim and Lundman [38]

Meaning unit	Condensed meaning unit	Subtheme	Theme
“It isn’t his [my brother’s] fault that I got sick. There were a lot of different factors contributing to that. He shouldn’t be thinking that, because … I’m not getting better even if he feels guilty, and he won’t feel better, either. So, I’m just trying to make him understand that it isn’t his fault.”	Several factors contributed to the patient’s illness. Nobody gets better when someone feels guilty.	Strong emotions	Being on a roller coaster
“Yes, or the focus became that I didn’t want to have too much attention for being sick, but only because I also thought that it was important for them [my family] to be taken care of, because I saw how much they were struggling with me being sick.”	Patients did not want too much attention for being sick. The family cared about the patient but were also having a difficult time.	Stronger attachments	Being in the same boat
“Yes, it [the ED treatment] started something good between us, as well as with my brother. I believe that we learned to talk together about difficult things. It’s not … as scary as it might seem. I’ve actually become more open with my brother, even though he wasn’t there.”	The ED treatment started something good. The siblings were able to talk to each other, and it wasn’t that scary.	Open communication	Being in the same boat

## Results

Most patients lived at home with their family during their ED sickness, and the ages of the patients at interview-time ranged between 20 and 38 years old (as seen in Table 1).

**Table 3:** Overview of themes, subthemes, and categories

Theme	Subtheme	Categories
Being on a roller coaster	Strong emotions	Worrying
		Feelings of guilt
	Ups and downs in life	Diagnoses and EDs
		How they are doing today
		Triggers
		Coping strategies
Being in the same boat	Stronger attachments	Including family
		Family members
		Support
	Open communication	Sharing with others
		New perspectives

### Theme: being on a roller coaster

#### Subtheme: strong emotions

Strong emotions such as worries, frustration, anger, discomfort, and desperation were experienced in the families during the illness period. Support and help from family and professionals were useful in that phase, and they led to greater trust and deeper mutual understanding. Patients experienced feelings of guilt about their parents not prioritizing their own health and lives and needing to take sick leave, leading patients to feel as though they were burdens on their families. Patients expressed that while family members—particularly parents—were concerned about their health, they often felt that dynamics in the family became characterized by frustration and anger (see Table 3 for details). Although parents tried to be caring, they often expressed this through anger and being overly controlling. Some patients noted that during treatment, they and their families were able to let their guard down and develop higher levels of trust through deeper mutual understanding; in contrast, others experienced discomfort and desperation during the illness period. In addition, parents often experienced feelings of guilt, leading them to deprioritize themselves, their health, and their lives during the illness period; such a toll often resulted in sick leave for parents. As mentioned above, this only caused the patients to feel more guilt at the thought that their ED was affecting their parents. One patient reflected on their parents during the illness period as follows:

I think that they [my parents] felt hopeless and kind of … took a sick leave during the time I was sick, to take care of me when I was at home. It lasted half a year, and they were struggling a lot, struggling with sleep, and I think that they were really sad and felt a great deal of guilt. (Participant 7)

Patients felt as though they were a burden on their families, which was followed by thoughts about deserving to be in pain. Listening to family members’ thoughts about their ED was especially painful for patients, some of whom also sensed that their parents were withholding their feelings to spare their sick daughter.

#### Subtheme: ups and downs in life

Life with an ED was described as a roller coaster, with participants sharing a wide range of ups and downs in their life. This roller coaster ranged from the high complexity of ED and mental illness comorbidity (especially anxiety and depression) to EDs in mothers and sisters to overcoming the diagnosis as an empowering experience.

Many siblings found it difficult to understand why their sister had become ill with an ED, despite having similar childhoods:

So, you can say, I think it’s a bit … I’m not going to say it’s weird that she [my sister] has been struggling with EDs, but we’re like a—what can I say? —a really normal, standard Norwegian family. (Participant 4, sibling)

At the time of data collection, three patients were still coping with their ED, receiving treatment, and living with their families. Others were doing better—living their lives and studying or working, with healthier relationships with their bodies, food, weight, and exercise. Patients who were still sick expressed the desire for a normal life; however, after a longstanding ED, which was often combined with anxiety and depression, these patients described it as being part of their life.

Some described the experience of having an ED and managing to overcome the diagnosis as empowering them to do what they wanted in life:

It has given me an attitude toward life in general, that things will work out. And that I can achieve what I want if I put in the effort. Things that used to feel quite scary and bad, don’t feel as bad now because I have kind of been at rock bottom, I have been really, really at rock bottom. So now, if something bad happens, it bounces off me a bit faster. The worst thing that has happened to you, is the worst thing that has happened to you. So, my threshold for what’s bad isn’t as low, it’s a bit higher in a way. (Participant 6)

**Fig. 1:**
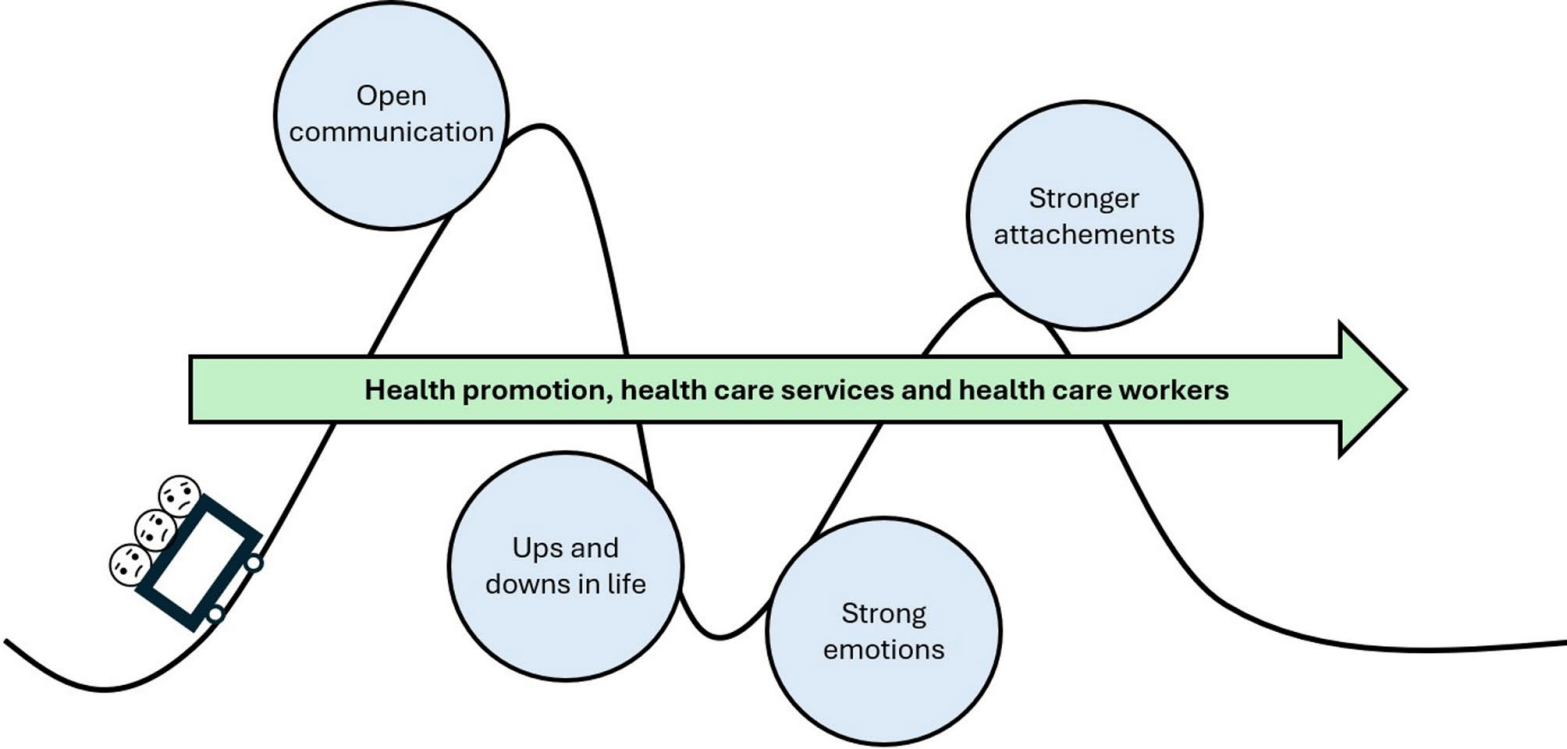
A roller coaster of emotions: Life with an ED is like a roller coaster, affecting the emotions of both patients and family

Many patients expressed that being treated for their EDs adversely affected their studies, which further contributed to the feeling of being on a roller coaster (Fig. 1). Those who were unable to continue with their education noted that they watched their friends’ and families’ lives continue, while they felt they were standing still. This created feelings of discontent in their lives that they were not where they wanted to be in life, and they often increasingly leaned on their families for support. Some patients developed strong feelings of guilt because they needed more support from their parents than their siblings to be able to get an education:

I’ve always fallen behind and not gotten an education because it [my ED] has taken up all my time, and Mom and Dad have needed to help me more, so I feel like … they [my siblings] are firmly established, with kids and a house and the whole package. It feels as if they have the perfect cookie-cutter life. Everything is in place, whereas I’m the one … “We help her because she can’t handle life.” I can still feel it. (Participant 2)

Many patients expressed their hopes of being able to move out of their parents’ home for their education, as they often expressed that being able to study gave them increased self-esteem. However, being a student also involved many new challenges, such as new routines with food and independent living, among other transitions in life. In addition, others expressed that moving away from their parents to a different town was an important factor in recovering from their ED—not to avoid their families, but to be able to get a fresh start somewhere where nobody knew about their ED:

But at the same time, I was really like…. When I first started studying, I wanted to move as far away as possible. To get to a completely new… new environment, and almost like starting from scratch, in a way, because I moved here in 2022, so it was right after I had finished the treatment I had been in. (Participant 8)

### Theme: being in the same boat

#### Subtheme: stronger attachments

Our results additionally revealed closer, more open, and stronger relationships in families after the experience of an ED. Stronger attachment involved a feeling of solidarity in the family while undergoing the illness period together. In that sense, a close relationship was understood as a relationship based on trust, support, and emotional attachment. Families with close relationships before the ED seemed to cope with the disease better, and patients from those families were faring relatively well at the time of data collection. One sibling described the relationships in their family after the ED as follows:

In our family, there’s always been openness about it [my sibling’s ED] since the beginning. And we—I—felt that we got even more attached to each other after the illness period. (Participant 3, sibling)

Including family members in both the ED treatment and the illness process seemed to be crucial, as EDs affected family life in terms of both relationships and communication. Some participants compared the ED to a new family member coming into the home and making life and relationships more difficult for the family during the illness period:

No, I found it really difficult, and I still do. Actually, it was like a new family member moved in. (Participant 5)

Patients expressed concerns about fighting with family members and their family’s dissolution, and they noted that such situations were often influenced by a sense of hopelessness, vulnerability, and internal conflicts in the family. In addition, while not representative of all families, some patients reported that their fathers did not take as central a role in their illnesses as their mothers:

The ED diagnosis affected the whole family at home, both siblings and parents. Most people living with EDs have struggled a lot at home over the years before being admitted for inpatient care. It makes the relationships in the family very different. I was experiencing the same thing, which is partly why I’m not living with my father anymore. Because, simply put, we don’t have a bad relationship, but it hasn’t worked out. Since I became sick, it’s gotten strict and very much like—I’ve moved on in the way that I see how I’m struggling and want to become healthy. I have a desire to live a life without being dependent on structure and food and routines above all. But my father’s sort of still trapped in an old pattern, afraid that I’ll get worse, and he’s afraid that I won’t eat on purpose, afraid of a lot. I don’t feel that he’s gotten over it, and then it’s not working, the communication. (Participant 10)

Patients often described their EDs as a fixation that made it necessary to lie in order to hide the disease. More openness, increased understanding, and knowledge about EDs among both patients and their family members could counteract the lies and decrease the conflicts:

It’s like something that goes on in most families, that communication gets estranged. Like, when someone in the house is sick, then you kind of have to take gentle steps. You can’t ask the questions that you might be thinking of. There’s a lot of being afraid of making a mistake or saying something wrong. (Participant 10)

An increased understanding of each other’s feelings, stronger relationships, and more openness were all common phrases used to describe the experience of going through an ED. Many patients emphasized that, as difficult as their experiences with an ED were, they and their families emerged stronger:

I sort of see my relationship with my parents as a period before I got sick and the period after I got sick. It’s two different things. Now, we can talk about anything, and we’re not afraid of bringing things up. Because now we know we can talk about things in a good way. (Participant 6)

Siblings added that having a sister with an ED had been challenging. One concern was that the disease had limited the family from doing activities together, because the sick family member’s needs came first. Another concern was the difference in siblings’ needs based on age. While talking about important topics with family members could lead to a deeper understanding among both patients and their families, and while involving family members in the healing process was considered to be important, some patients in our study reported a concern about the ripple effect, where exposure to details about the ED might negatively impact their sisters. One patient described the treatment period in relation to her sister as follows:

I believe she felt left out, because there were also some important parts [of the illness] that she could have participated in … because I’d been comparing myself with her during that period. And because of that, I’m partly glad that she wasn’t included, because I was afraid, I remember, that my ED would affect her in some way, if she were to hear my thoughts and about how I was feeling: that she would pick up after me and that it would become a competition between us. (Participant 7)

When family members were involved in treatment together with the patients with an ED, a strong feeling of not being alone—i.e., of “being in the same boat”—was highlighted as being important for the family. Some patients described their mother and/or sister also having an ED. In cases where mothers had also had an ED in their youth, the mother–daughter relationship and communication were described as strong, as previous personal experiences with an ED could give the mother a deeper understanding of the illness, making it easier for her daughter to be open and to share:

Yeah, my mom had bulimia, but I had anorexia. So, there’s a difference. But, like, she understands me on a different level than others do. She understands that it’s not just about eating, it’s not just about getting better. It’s not like I can just press a button, and everything will be fine. She’s always understood that, and, um…Dad didn’t understand that I was sick when I was sick. But my mom understood almost right away. She could see it in me, she recognized it… (Participant 9).

When the ED patient was less than 18 years old, her parents were accountable for ensuring her needs; however, after the age of 18, patients were allowed to take control of their own needs. That transition was described by patients as being challenging for both patients and their parents: parents often struggled to understand their own role as supportive, for they truly wanted to help their sick adult daughter; however, having their sick daughter take responsibility for her own life lifted some weight off the parents’ shoulders. EDs also changed the dynamics in families in other ways. After experiencing the disease, both patients and family members felt less guilt and had more patience. Some patients did not want treatment but were worried about the consequences for the family dynamics and the level of conflict in their family if they refused. In any case, parental involvement at an early stage seemed to be an important contributor to receiving support:

In short, involving parents is important. Even if you don’t feel like it, it’s necessary when you’re at your sickest point. Or even if you don’t want to, it’s a crucial part of being able to continue. (Participant 8)

#### Subtheme: open communication

Sharing one’s experiences with others in the same boat as them was important, as it afforded new perspectives and could reduce feelings of guilt and shame. Sharing one’s own experiences seemed exclusively positive, even when it was challenging; it was described as rewarding, contributing to openness and an arena for establishing new bonds that could also lead to new friendships and a feeling of not being alone. Patients described how being able to share their thoughts about difficult topics with family members contributed to an increased acceptance of the ED. It also gave them insight into others in the same situation when similar problems were present in other families. This could lead to a reduction in feelings of guilt and shame, due to understanding that they were not to blame for becoming sick. Sharing also helped participants view things from new perspectives (Fig. 1) and relate to others, for an altogether stronger family.

Patients expressed that being honest with family members and not hiding their illness made it easier to seek help, as well as to give help. One patient explained her open communication with her younger brother along those lines:

He didn’t have to be afraid to ask me anything. There were no dangerous questions. If I didn’t feel like answering, then I could say so. But if he was wondering about something, he just needed to ask. And when he understood that more directly—like “Wow!”—he could ask anything. It all became more open, and we could talk about everything. So, it was really nice. (Participant 10)

Some patients experienced self-harm and overeating combined with ED as an expression of their feelings, in the same way as not eating. Not all patients wanted help or attention, and in those cases, sharing and communication were challenging. Looking at the ED from the family members’ perspectives was described as being painful but an important part of recovery and an essential part of the family’s attempt to have a better life together. Good communication and sharing contributed to less anger, fewer accusations, and less nagging. Furthermore, being able to bring up difficult topics and to support each other was important.

After families experienced an ED, it seemed that their communication had improved, and it was easier to talk about difficult topics—although it could still be hard to find the right words, because strong feelings of shame were attached to the ED. Therefore, the disease was described as a life event that forced deeper communication in the family. Relatedly, open communication was important to prevent misunderstandings:

When I was 14, I was little, and I started to think like, “What? This isn’t me.” But they were my thoughts; I was the one talking. And I was like, “What? Is there a monster inside me?” And I became scared, and my mother would say, “That’s not you talking. I know that it’s the ED.” And then I understood in advance that it was a way of defending me, of saying that they weren’t mad at me, their daughter: that they understood that it [something I said] wasn’t something that I meant but just a sick thought. … “We’re trying to talk to you, we’re not strict with you”—trying to spare me a little bit, you know. But for me, taking it literally, I was scared and like, “Oh my god—what am I feeling inside my body? There’s something eating me up inside. It’s like there’s a monster inside me.” (Participant 10).

Patients found it useful to be able to learn a language to express themselves, combined with trying to see themselves from the perspectives of their family members. Those new perspectives were a useful tool that helped them gain a better understanding about themselves, more easily accept their EDs, and be able to talk about hurtful topics instead of holding everything inside.

An open relationship and deeper understanding were described as follows:

…what should we say, an open relationship, maybe a bit deeper conversation in between, could talk a little more like that. Not just superficial, maybe more like diving into things, and that applies both to the eating disorder, but also to everything else going on in life. That we have more of those conversations… So, overall, it has kind of strengthened the relationship… (Participant 8).

## Discussion

We identified two main themes. *Being on a roller coaster* captured the feeling of the family living with all the highs and lows of EDs, and it included the subthemes *strong emotions* and *ups and downs in life*. *Being in the same boat* articulated the feeling of solidarity in the family while going through the illness period together, and it included the subthemes ‘*stronger attachments’* and ‘*open communication’* (Table 3).

Patients experienced feeling guilty about the ED affecting their family and about their parents not prioritizing their own health and lives. This might lead one to think that it would have been beneficial for the parents and siblings to ‘jump off the roller coaster’, namely setting boundaries that enabled them to take care of their own health and needs. Our findings are supported by the results of past studies showing the burden experienced by parents of children with EDs and the illness’s negative impact on everyday life and intrafamily relationships [21–24], including a lower quality of life for patients and family [17–20]. Our findings also suggest a lower quality of life for both patients and family in the illness period, which made patients feel as if they were burdening their families. This can be understood in relation to previous studies highlighting that families of patients with EDs need professional help to cope with their emotional reactions and to prioritize their own needs and the needs of other family members [19, 20, 23]. Similar to our study, results from another qualitative study found that family members of adults with EDs often face unique challenges and describe the treatment as “like a roller coaster” [26]. The fact that other studies have produced similar findings, even though they describe the treatment and not the family relationships (as we do), reinforces our current findings.

Life with an ED can be like a roller coaster, and patients often need more support from parents than their siblings do. Most of our patient participants lived with their parents, in line with earlier findings [14–16]. Our findings suggest that some families may be stuck in a pattern of unhelpful interactions that unintentionally contribute to the persistence of the EDs, as other studies have found [10–13], which could partly explain why some patients with EDs experience improvement after moving. Our results additionally show that being a sibling of someone with an ED can be challenging, because the illness affects the whole family. This result corresponds to findings from other studies showing that siblings report insufficient care and negative changes in family life because the illness consumes a considerable amount of time and space [27–29].

Our results show that the relationships and communication in families were negatively affected during the illness period, as discovered in other studies [4–6, 32]; however, our results also reveal closer, more open, stronger relationships in families after experiencing an ED. Such findings indicate hope and potential for growth for the families, and they are valuable knowledge from a clinical perspective, as practitioners can facilitate such growth processes and highlight the various benefits to be gained for families from such challenging journeys. The stronger relationships were partially explained by viewing an ED as a life event that forces deeper communication and understanding in the family; this understanding is likely to increase in both directions, affording new perspectives. Families who had close relationships before the illness seemed better equipped to help their sick family members, and patients seemed to navigate the disease in productive ways with the support of their families. This dynamic can be partially explained as family support; however, there is probably a complex mix of different positive factors at work.

Some participants described the relationships in their families before and after EDs as two different things; this could be explained by several factors, such as the family’s inclusion in the illness period, the increased understanding for each other, and more openness. This aligns with the findings from another study, where parents’ aspirations shifted from fixing the ED to prioritizing a relational goal of unrelenting connection with their child [25]. When family members had a shared understanding of a problem, it seemed that the threshold for conflicts and fighting was higher, thereby offering more room for dialogue between family members. These results are similar to the findings of other studies, in which including the family in therapy has proven to be helpful [17, 19, 20, 37, 46, 47].

Fear of conflict was apparent in some families; moreover, some fathers did not take as central a role in responding to the illness as mothers, as another study has shown [48]. Such a situation can be difficult for both daughters and fathers, and some may need distance in their relationships as a result. There may be a potential for recovery in the father–daughter relationships in some families, and the role of fathers might be underexplored. If fathers are ensured to become a source of support for daughters with ED, the increased support could positively affect family relationships and bring about healing from illness.

Because an ED is often a nonverbal expression of underlying difficulties [49], being able to share difficult emotions can help patients cope with them. This dynamic could also lead to a deeper understanding of the ED for both patients and families, as well as strengthen the feeling of solidarity.

Further research on EDs should focus on the implications of EDs for intrafamily relationships and should gather the voices of other vulnerable groups, including students, men/boys, other types of patients, and other family members. Research on gender differences is also required to better understand changes in EDs.

### Strengths and limitations

Our study had several strengths, such as its inclusion of the voices of young adults with EDs, of their siblings, and of a co-researcher. At the same time, the onset of ED for some participants was more than a decade prior to the study, which made it difficult for them to remember details about the illness. However, the retrospective view in our study might also be seen as a strength, because patients with an active ED can find it difficult to engage in conversation while they are sick [20]. An additional strength of the follow-up design, in which patients and siblings were interviewed years after illness, could be that it affords a new perspective and new important knowledge—namely, on how participants reflect on their family relationships years after their ED and treatment—and provides insight into how families are doing after the illness period. Conducting interviews either face-to-face or virtually also seemed to be a strength, as it enabled participants to choose what they preferred and lowered the threshold to participate. Furthermore, as authors, we all brought different perspectives to the research: RAS brought the patient’s perspective; JLK and BSB brought experience with working and conducting research in the field over several years; GH brought research experience as well as clinical competence; and LMJ added a new perspective. To our knowledge, this study was the first to investigate the experiences concerning family relationships of adult patients with AN and their siblings, who reflect on their experiences as emerging adults.

Nevertheless, some limitations should be kept in mind. For one, our sample of patients lacked the voices of men/boys with EDs, and only two siblings participated. Because of small sample size of siblings, data saturation was not reached, which might limit the breadth and depth of the results. It would have been preferable to have included a higher number of siblings. Due to ethical considerations, we could not contact siblings directly, meaning the patients had to recruit siblings for us, which made recruitment more difficult. Even so, we believe that the voices of the siblings who were included are of high importance in the study. Additionally, there may have been selection bias, because our sample included only individuals who have (partially) overcome their EDs and therefore have the interest and resources to participate, in contrast to patients with ED who did not participate in the interviews. Furthermore, if we chose to use a thematic analysis instead of qualitative content analysis, we might have achieved more depth in our results. Finally, un this study, mostly mothers are mentioned, and some parents (not necessarily only fathers) did not engage as much in their daughter’s illness. Mother–daughter and father–daughter relationships are different relationships, and this must be kept in mind when interpreting the results.

## Conclusions

Our data revealed two main themes. First, the metaphor of *being on a roller coaster* captured the feeling of the family living with all the highs and lows of EDs. Second, the metaphor of *being in the same boat* articulated the feeling of solidarity in the family while going through the illness period together.

The burden of providing care for patients with EDs can be immense and can negatively impact daily life and family relationships. Family relationships affect patients, and with the intense pressure that EDs bring, it is crucial to ensure that families exert a positive influence and avoid unhelpful interactions, which may prolong the illness. Help and support for coping with EDs should be prioritized for both adult patients and their families.

## Supplementary Information

Supplementary Material 1.

Supplementary Material 2.

## Data Availability

The data collected and analyzed are not available due to ethical considerations, maintaining the participants’ anonymity. The corresponding author may be contacted about the data upon reasonable request.

## Abbreviations

AN: Anorexia nervosa
BN: Bulimia nervosa
ED: Eating disorder
MFT: Multi family therapy
