# Supplementary material for: Experiences with family relationships following eating disorders: a roller coaster of emotions
Source: BMC Psychiatry. 2025 Oct 27;25:1023. doi: 10.1186/s12888-025-07422-x (PMC12557936; doi:10.1186/s12888-025-07422-x)
Supplement: Supplementary file 2 — Supplementary Material 2. [file 12888_2025_7422_MOESM2_ESM.docx]

**Interview Guide for Siblings**

**Introduction and Information**
Information about the project and presentation of the researcher. Information about confidentiality and complete anonymity. Emphasize that the main focus is on the participants and their experiences with multi family therapy (MFT).

**Questions:**
• Who are the members of your family, their ages, current living situation, and your sister’s diagnosis?
• Can you describe your experience of participating in MFT?
• What did you do?
• Were there any challenges in participating as a sibling in MFT?
• Who from your family participated in MFT?
• What do you think the members of your family think about participating in MFT?
• How has participating in MFT affected you and your relationship with your family?
• Did you learn any coping strategies in MFT?
• Have there been any challenges in your family related to participating in MFT?
• If yes, what are your coping strategies for dealing with them?
• Did any of your coping strategies change after MFT?
• What does MFT mean to you?
• What do you take away from participating in MFT?
• Would you recommend MFT to others? If so, why?

**Extra questions if there is enough time:**
• What was good/what was significant?
• How was it meeting other patients/families?
• How were the therapists?
• What was bad?
• Do you know any participants who were not satisfied? With what?
• Have you participated digitally, in the long or short version of MFT (experiences with this)?
• How are you feeling now?

• Is there anything important I forgot to ask about?

**Summing up and ending:** Ensure the informant has said everything they intended to. Check how the informant is feeling emotionally and ensure that there is someone available to talk to after the interview if there is a need to address difficult emotions.
